# Postoperative delirium after pharyngolaryngectomy with esophagectomy: a role for ramelteon and suvorexant

**DOI:** 10.1007/s10388-017-0570-z

**Published:** 2017-01-21

**Authors:** Eisuke Booka, Yasuhiro Tsubosa, Teruaki Matsumoto, Mari Takeuchi, Takashi Kitani, Masato Nagaoka, Atsushi Imai, Tomoyuki Kamijo, Yoshiyuki Iida, Ayako Shimada, Katsushi Takebayashi, Masahiro Niihara, Keita Mori, Tetsuro Onitsuka, Hiroya Takeuchi, Yuko Kitagawa

**Affiliations:** 1 Division of Esophageal Surgery, Shizuoka Cancer Center Hospital, 1007 Shimonagakubo, Nagaizumi-cho Sunto-gun, Shizuoka, 411-8777 Japan; 2 Division of Psycho-Oncology, Shizuoka Cancer Center Hospital, Shizuoka, Japan; 3 Palliative Care Center, Keio University Hospital, Tokyo, Japan; 4 Division of Head and Neck Surgery, Shizuoka Cancer Center Hospital, Shizuoka, Japan; 5 Clinical Trial Coordination Office, Shizuoka Cancer Center Hospital, Shizuoka, Japan; 6 Department of Surgery, Keio University School of Medicine, Tokyo, Japan

**Keywords:** Pharyngolaryngectomy with esophagectomy, Postoperative delirium, Minor tranquilizers, Ramelteon, Suvorexant

## Abstract

**Background:**

Postoperative delirium is common after extensive surgery, and is known to be associated with sleeping medications. In this study, we aimed to investigate the relationships between sleeping medications and postoperative delirium after pharyngolaryngectomy with esophagectomy.

**Methods:**

We performed a retrospective analysis of 65 patients who underwent pharyngolaryngectomy with esophagectomy at Shizuoka Cancer Center Hospital between January 2012 and March 2016. All data were assessed by two psychiatrists, and univariate and multivariate analyses were performed.

**Results:**

Postoperative delirium developed in 9 (13.8%) patients, with most cases (77.8%) occurring between postoperative day (POD) 1 and POD 3. Of the 24 patients taking a minor tranquilizer after surgery, 8 (33.3%) became delirious, but, of the remaining 41 patients taking ramelteon with or without suvorexant, only one (2.4%) became delirious after surgery. Moreover, of the 16 patients taking both ramelteon and suvorexant, no postoperative delirium was observed. Ramelteon with or without suvorexant was significantly associated with a decreased rate of postoperative delirium compared with minor tranquilizer use (*p* = 0.001). Multivariate analysis confirmed that the use of ramelteon with or without suvorexant was the only significant preventive factor of postoperative delirium (odds ratio 0.060, *p* = 0.013).

**Conclusion:**

The use of ramelteon with or without suvorexant was the only significant preventive factor of postoperative delirium after pharyngolaryngectomy with esophagectomy. However, using minor tranquilizers was associated with postoperative delirium. We recommend ramelteon with or without suvorexant for preventing postoperative delirium after pharyngolaryngectomy with esophagectomy.

## Introduction

Delirium is defined as a change in mental status caused by physical disorders, and it is characterized by disturbed consciousness, reduced ability to focus, and changed cognitive function [1, 2]. Indeed, delirious patients are often disorientated in time, place, and person. Based on clinical presentation, delirium has been classified into three motor subtypes: hyperactive/agitated, hypoactive/somnolent, and mixed; the latter of these fluctuates between the hyperactive and hypoactive types during the course of the illness [1, 2].

Postoperative delirium is a common and serious complication after extensive surgery. However, there are important pre-, peri-, and postoperative factors associated with the risk of its development [1]. We previously reported the incidence and risk factors for postoperative delirium after major surgery for head and neck cancer [1]. In that study, we concluded that most cases of postoperative delirium occurred between postoperative day (POD) 1 and POD 3. An associated multivariate analysis revealed that being older than 70 years was a significant risk factor for postoperative delirium [1].

Pharyngolaryngectomy with esophagectomy has been shown to be a highly invasive surgery, following which patients cannot speak because of the removal of the larynx [1, 3, 4]. We considered that the patients were at particularly high risk of developing postoperative delirium after this surgery because of their inability to express feelings verbally [1]. Moreover, patients often complain of insomnia after such extensive surgery, and sleeping medications are frequently needed [5]. Of the therapeutic options, it has been reported that minor tranquilizers promote the development of postoperative delirium. By contrast, ramelteon, which is an agonist of melatonin approved for the treatment of insomnia characterized by difficulty at sleep onset by the US Food and Drug Administration, has been associated with reduced rates of postoperative delirium [5, 6]. Suvorexant, which is a dual orexin receptor antagonist that was approved in late 2014 in the US and Japan for the treatment of insomnia characterized by difficulty achieving and/or maintaining sleep, could also have similar beneficial associations with postoperative delirium, but this has not been studied to date [7].

We hypothesized that combination therapy with ramelteon and suvorexant could prevent postoperative delirium after pharyngolaryngectomy with esophagectomy. In this study, we, therefore, aimed to investigate the relationships between sleeping medications and postoperative delirium after pharyngolaryngectomy with esophagectomy.

## Patients and methods

### Patients

We retrospectively analyzed the records of 65 patients who underwent pharyngolaryngectomy with esophagectomy at Shizuoka Cancer Center Hospital, Shizuoka, Japan. All patients undergoing the procedure between January 2012 and March 2016 were included. The study was conducted with the approval of the Ethics Committee of Shizuoka Cancer Center Hospital.

### Surgical procedure

Pharyngolaryngectomy with esophagectomy was performed by esophageal or head and neck surgeons based on tumor location. Free jejunal transfer with microvascular anastomosis was performed to repair the cervical defect between the hypopharynx and the oral side of the remnant esophagus by plastic and reconstructive surgeons in all cases [3]. Two anastomoses were required: pharyngojejunal and jejunal-esophageal.

#### Postoperative management

All patients were managed in the intensive care unit immediately after surgery, and received mechanical ventilation under propofol anaesthesia. All patients were extubated on POD 1 and then admitted to the general surgical ward. For postoperative analgesia, fentanyl was provided through an intravenous catheter and ropivacaine hydrochloride hydrate was provided through an epidural catheter. Enteral nutrition was initiated on POD 1 via a feeding tube in all cases. All patients received sleeping medications via a feeding tube from POD 1 to discharge to postoperatively treat insomnia. Because delirium developed 1 week postoperatively in our previous study, sleeping medications were administered throughout the duration of the hospital stay in this study [1]. Sleeping medications consisted of minor tranquilizers (mainly zolpidem), ramelteon (8 mg), and suvorexant (20 mg). Patients received minor tranquilizers between January 2012 and October 2013, ramelteon only between November 2013 and February 2015, and ramelteon with suvorexant between March 2015 and March 2016. When we changed the sleeping medications from minor tranquilizers to ramelteon, some patients suffered from insomnia, so we added suvorexant to ramelteon. If patients complained of insomnia even after taking sleeping medications, tetracyclic antidepressants were added via a feeding tube and intravenous infusion of a tranquilizer, such as haloperidol, was not administered.

#### Data collection methods

A retrospective chart review was used to collect data. Preoperative data included age, gender, body mass index, medical illness history, psychiatric illness history, smoking history (>10 cigarettes daily for ≥2 years), alcohol consumption (estimated weekly ethanol intake of ≥200 mL), Eastern Cooperative Oncology Group (ECOG) performance status, and American Society of Anesthesiologists physical status. Perioperative data included tumor location, operation duration, blood loss, urinary output, amount of blood transfused, infusion volume, and maximum body temperature after surgery. Postoperative data included the types of sleeping medications used.

Delirium was diagnosed according to the criteria in the fifth edition of the Diagnostic and Statistical Manual of Mental Disorders, published by the American Psychiatric Association [8]. All patients’ charts, which had been logged by surgeons, psychiatrists, and nurses, were systematically reviewed by two psychiatrists (T. M. and M. T.). The methods used were the same as in our previous study [1]. Data were collected from the day of admission to POD 14. We included hyperactive delirium and mixed type delirium, both of which were important during the postoperative management; however, hypoactive delirium was not so important during the postoperative management and was excluded.

#### Statistical analysis

The statistical analysis was performed using IBM SPSS, Version 23.0 (IBM Corp., Armonk, NY, USA). Demographic and surgical data (pre-, peri-, and postoperative) were compared between patients with and without delirium. Categorical data were analyzed using Fisher’s exact test or the *χ* ^2^ test, as appropriate. Quantitative data were analyzed using Mann–Whitney’s *U* test.

A *p* value of <0.05 was considered statistically significant for all analyses, and variables that achieved significance in the univariate analysis were entered into the multivariate analysis for estimation of the preventive factor of postoperative delirium. We then used multiple logistic regression analysis to adjust for multiple preventive factors and interactions, and estimated the goodness-of-fit using Hosmer–Lemeshow’s test.

## Results

### Patient characteristics

The 65 study participants comprised 56 men and 9 women, and the median age was 66.0 (range 47–88) years. The demographic and perioperative data are shown for all patients in Tables 1 and 2. The most common tumor location was the hypopharynx (86.2%).

**Table 1:** Baseline data for patients with and without delirium

Variable	All patients	Delirium (+)	Delirium (−)	*p* value
Total	65	9	56	
Age (years)	66.0 (61.5, 72.0)	69.0 (65.5, 79.0)	66.0 (61.0, 72.0)	0.128
Age category, *n* (%)				0.769
<70	39 (60.0%)	5 (55.6%)	34 (60.7%)	
≥70	26 (40.0%)	4 (44.4%)	22 (39.3%)	
Gender, *n* (%)				0.433
Male	56 (86.2%)	7 (77.8%)	49 (87.5%)	
Female	9 (13.8%)	2 (22.2%)	7 (12.5%)	
Body mass index (kg/m^2^)	19.6 (18.1, 21.6)	18.2 (16.1, 21.3)	19.9 (18.2, 21.7)	0.323
Medical history, *n* (%)				
Hypertension	18 (27.7%)	3 (33.3%)	15 (26.8%)	0.684
Diabetes mellitus	10 (15.4%)	0 (0%)	10 (17.9%)	0.168
Hyperlipidemia	2 (3.1%)	1 (11.1%)	1 (1.8%)	0.133
Stroke	8 (12.3%)	1 (11.1%)	7 (12.5%)	0.906
Coronary heart disease	3 (4.6%)	0 (0%)	3 (5.4%)	0.477
Renal dysfunction	2 (3.1%)	0 (0%)	2 (3.6%)	0.565
Malignant tumor	22 (33.8%)	2 (22.2%)	20 (35.7%)	0.427
Psychiatric disorder	4 (6.2%)	1 (11.1%)	3 (5.4%)	0.505
Alcoholism, *n* (%)^a^	51 (78.5%)	6 (66.7%)	45 (80.4%)	0.354
Chronic smoking, *n* (%)^b^	58 (89.2%)	9 (100.0%)	49 (87.5%)	0.262
Performance status (Eastern Cooperative Oncology Group)				0.022
0	37 (56.9%)	2 (22.2%)	35 (62.5%)	
1	21 (32.3%)	4 (44.4%)	17 (30.4%)	
2	7 (10.8%)	3 (33.3%)	4 (7.1%)	
Physical status (American Society of Anesthesiologists)				0.798
Grade 1	10 (15.4%)	1 (11.1%)	9 (16.1%)	
Grade 2	45 (69.2%)	6 (66.7%)	39 (69.6%)	
Grade 3	10 (15.4%)	2 (22.2%)	8 (14.3%)	

Data are shown as median (interquartile range) or number (percent)

^a^Alcoholism was defined as drinking an estimated weekly ethanol intake of ≥200 mL

^b^Chronic smoking was defined as smoking >10 cigarettes daily for at least 2 years

**Table 2:** Intraoperative and postoperative data in patients with and without delirium

Variable	All patients	Delirium (+)	Delirium (−)	*p* value
Total	65	9	56	
Tumor location, *n* (%)				0.873
Oropharynx	3 (4.6%)	0 (0%)	3 (5.4%)	
Hypopharynx	56 (86.2%)	8 (88.9%)	48 (85.7%)	
Larynx	4 (6.2%)	1 (11.1%)	3 (5.4%)	
Major salivary gland	1 (1.5%)	0 (0%)	1 (1.8%)	
Thyroid	1 (1.5%)	0 (0%)	1 (1.8%)	
Operation time (min)	573.0 (522.5, 652.5)	553.0 (407.0, 696.0)	576.0 (526.0, 652.8)	0.642
Blood loss (mL)	410.0 (289.0, 564.5)	442.0 (310.0, 776.5)	410.0 (283.5, 535.8)	0.447
Urinary output (mL)	740.0 (482.5, 1115.0)	760.0 (447.5, 1160.0)	730.0 (492.5, 1117.5)	0.894
Blood transfusions (mL)	0 (0, 0)	0 (0, 655.0)	0 (0, 0)	0.334
Volume of infusion (mL)	4200.0 (3525.0, 5425.0)	3500.0 (2960.0, 6200.0)	4245.0 (3650.0, 5400.0)	0.352
Maximum body temperature after surgery (°C)	38.1 (37.8, 38.6)	38.1 (37.7, 38.9)	38.1 (37.8, 38.5)	0.805
Sleeping medication, *n* (%)				0.002
Minor tranquilizer	24 (36.9%)	8 (88.9%)	16 (28.6%)	
Ramelteon only	25 (38.5%)	1 (11.1%)	24 (42.9%)	
Ramelteon and suvorexant	16 (24.6%)	0 (0%)	16 (28.6%)	

Data are shown as median (interquartile range) or number (percent)

### Occurrence of postoperative delirium

**Fig. 1:**
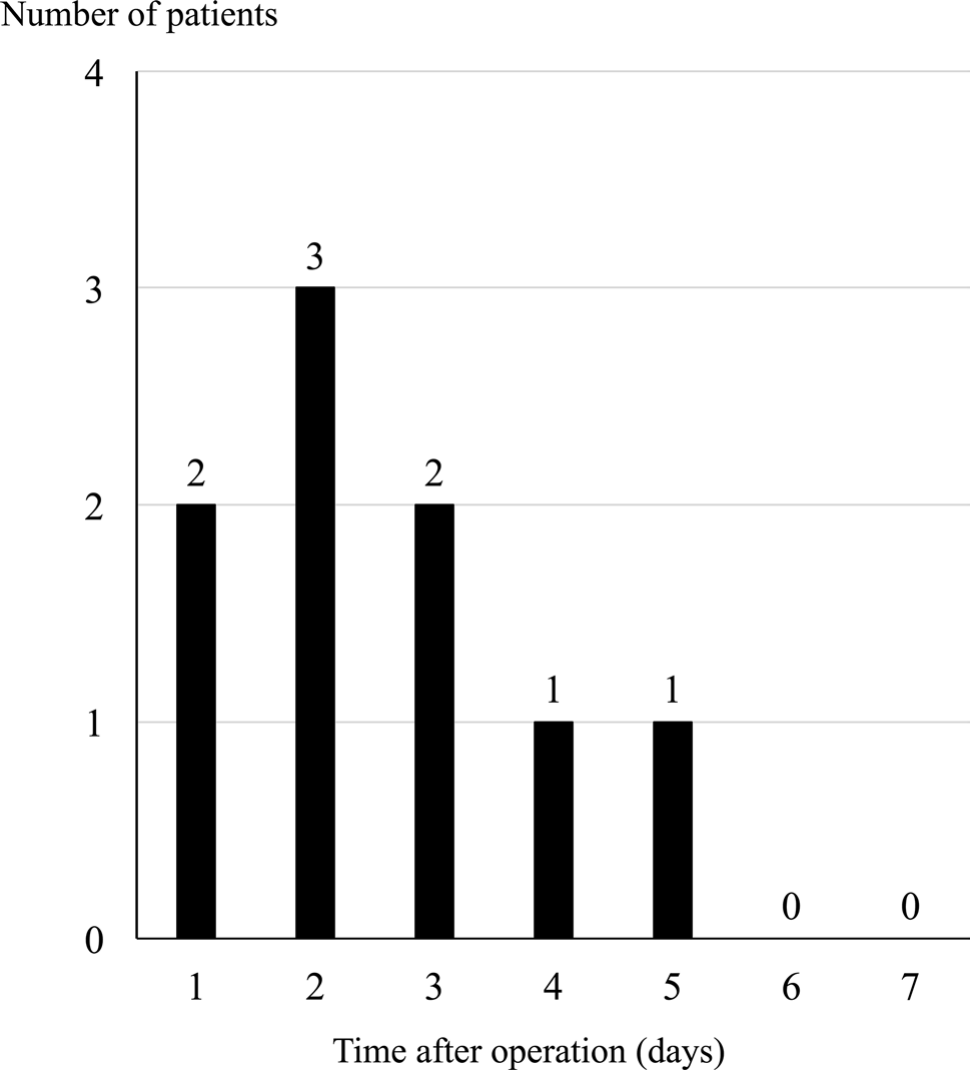
Time of postoperative delirium onset

Among the 65 patients included in the study, postoperative delirium was diagnosed in 9 (13.8%), and although all of these cases were observed between POD 1 and POD 5, most (77.8%) were observed between POD 1 and POD 3 (Fig. 1). Of the 24 patients taking minor tranquilizers after surgery, 8 (33.3%) developed postoperative delirium. By contrast, of the other 41 patients taking ramelteon with or without suvorexant, only 1 (2.4%) developed postoperative delirium after surgery; moreover, of the 16 patients taking both ramelteon and suvorexant, no cases of postoperative delirium occurred. Therapy with ramelteon with or without suvorexant was associated with a significantly decreased frequency in postoperative delirium compared with minor tranquilizer use (*p* = 0.001). Although no postoperative delirium occurred when suvorexant was administered with ramelteon, there was no significant additive effect of adding suvorexant to ramelteon to prevent postoperative delirium (*p* = 0.418). However, ramelteon only was not sufficient for insomnia, and suvorexant made up for the deficiency.

### Univariate and multivariate analyses of preventive factors for postoperative delirium

Univariate analyses revealed that the ECOG performance status and the use of ramelteon with or without suvorexant were the preventive factors of postoperative delirium after pharyngolaryngectomy with esophagectomy. Using these as preventive factors, the subsequent multivariate logistic regression analysis showed that the use of ramelteon with or without suvorexant remained the only significant preventive factor of postoperative delirium (odds ratio 0.060) (Table 3). The goodness-of-fit of this model was good from the result of Hosmer–Lemeshow’s test (*p* = 0.914). The use of minor tranquilizers was associated with the development of postoperative delirium.

**Table 3:** Multivariate analysis of preventive factors for postoperative delirium

Variable	*p* value	Hazard ratio	95% CI
Ramelteon with or without suvorexant	0.013	0.060	0.0066–0.55
ECOG performance status	0.186		

*CI* confidence interval, *ECOG* eastern cooperative oncology group

## Discussion

In a previous study by our group, postoperative delirium developed in 50 of 293 patients (17.1%) after surgery for head and neck cancer, and we did not consider the impact of sleeping medication on the development of postoperative delirium [1]. However, other research has shown that the use of minor tranquilizers was associated with the development of postoperative delirium and that ramelteon might prevent postoperative delirium [5, 6]. In this study, we, therefore, investigated the association of sleeping medications with the development of postoperative delirium.

We showed that eight of the 24 patients (33.3%) taking minor tranquilizers after surgery developed postoperative delirium, but that only one of the 41 patients (2.4%) taking ramelteon with or without suvorexant developed postoperative delirium. In addition, there were no cases of postoperative delirium among the 16 patients taking both ramelteon and suvorexant. These results indicate three possibilities. First, that the use of minor tranquilizers caused the development of postoperative delirium. Second, that ramelteon, suvorexant, or combination therapy with both drugs decreased the development of postoperative delirium. Third, that both the first and second conclusions are correct. All patients were administered sleeping medications postoperatively and there was a lack of control group receiving placebo in this study, so we cannot comment on the frequency of postoperative delirium in those not administered sleeping medications. However, compared with our previous study [1], patients taking minor tranquilizers after surgery did develop postoperative delirium more often, while those taking ramelteon with or without suvorexant developed postoperative delirium less often. Thus, there is a high possibility that the third possibility is correct, and that at the very least, ramelteon with or without suvorexant was associated with a lower risk of producing delirium compared with minor tranquilizers.

Minor tranquilizers have been associated with delirium in most previous studies, with research showing reduced use after surgery and for old patients [2, 9, 10]. By contrast, a randomized, placebo-controlled trial has shown that ramelteon can help prevent delirium [6]. In vitro studies have also demonstrated that ramelteon has 6- and 3-fold higher affinities for melatonin receptors 1 and 2, respectively, when compared with melatonin [6]. This suggests that a reduced frequency of delirium might be associated with higher affinities for these receptors, supporting a possible pathogenic role for melatonin neurotransmission in delirium [6]. Suvorexant is a dual orexin receptor antagonist that is used to treat insomnia characterized by difficulty achieving and/or maintaining sleep, but no report has been published on whether it can prevent delirium [7]. Nevertheless, similar to the implicated role of melatonin, there is a possible pathogenic role for disordered orexin neurotransmission in delirium.

It cannot be derived from this study whether ramelteon or suvorexant produced a lower risk for developing postoperative delirium or whether they actually prevented the condition. If ramelteon and suvorexant were only associated with a lower risk for developing delirium, we should only recommend their use in patients suffering from insomnia. However, if ramelteon and suvorexant can actually prevent postoperative delirium, we should consider administering them to all patients at high risk of developing postoperative delirium, regardless of whether or not they suffer from insomnia. In our previous study, an age of >70 years (odds ratio 3.935) was a significant determinant for postoperative delirium [1].

In this study, although there was no additive effect when suvorexant was given with ramelteon to prevent postoperative delirium, it was notable that there were no cases of postoperative delirium in any of the patients taking both medications together. Moreover, ramelteon alone was not sufficient to treat insomnia and suvorexant made up for the deficiency. It is possible that combination therapy with ramelteon and suvorexant had a stronger preventive effect on postoperative delirium. To investigate whether ramelteon is sufficient alone or in combination with suvorexant to prevent postoperative delirium, a randomized controlled trial is needed.

There are two important limitations of our study. First, we used a retrospective design at a single institution, and it is possible that the diagnosis was not accurate. Second, we did not consider the length of sobriety or use of sleeping medications before surgery. Nevertheless, all patients’ charts were systemically assessed by two psychiatrists to overcome some of these biases.

In conclusion, we showed that ramelteon with or without the use of suvorexant was associated with fewer cases of postoperative delirium after pharyngolaryngectomy with esophagectomy, but minor tranquilizers were associated with the development of postoperative delirium. We recommend that treatment with ramelteon, with or without suvorexant, be used instead of minor tranquilizers for patients undergoing pharyngolaryngectomy with esophagectomy after surgery to prevent postoperative delirium.

## References

[1] Booka E, Kamijo T, Matsumoto T (2016). Incidence and risk factors for postoperative delirium after major head and neck cancer surgery. J Craniomaxillofac Surg.

[2] Takeuchi M, Takeuchi H, Fujisawa D (2012). Incidence and risk factors of postoperative delirium in patients with esophageal cancer. Ann Surg Oncol.

[3] Booka E, Tsubosa Y, Niihara M (2016). Risk factors for complications after pharyngolaryngectomy with total esophagectomy. Esophagus..

[4] Fujiki M, Miyamoto S, Sakuraba M (2015). Risk factors for tracheal necrosis after total pharyngolaryngectomy. Head Neck.

[5] Marcantonio ER, Juarez G, Goldman L (1994). The relationship of postoperative delirium with psychoactive medications. JAMA.

[6] Hatta K, Kishi Y, Wada K (2014). Preventive effects of ramelteon on delirium: a randomized placebo-controlled trial. JAMA Psychiatr.

[7] Winrow CJ, Gotter AL, Cox CD (2011). Promotion of sleep by suvorexant-a novel dual orexin receptor antagonist. J Neurogenet.

[8] American Psychiatric Association (2013). Diagnostic and stastical manual of mental disorders.

[9] Francis J, Martin D, Kapoor WN (1990). A prospective study of delirium in hospitalized elderly. JAMA.

[10] Rogers MP, Liang MH, Daltroy LH (1989). Delirium after elective orthopedic surgery: risk factors and natural history. Int J Psychiatry Med.

